# Carotid Plaque Characteristics Evaluation on DUS and MDCTA: Interobserver and Intermodality Agreement in a Single-Center Study

**DOI:** 10.3390/medicina62040724

**Published:** 2026-04-10

**Authors:** Perica Mutavdzic, Tijana Kokovic, Branko Gakovic, David Matejević, Ivan Tomić, Miloš Sladojević, Aleksandar Tomic, Igor Koncar

**Affiliations:** 1 Faculty of Medicine, University of Belgrade, 11000 Belgrade, Serbia; mutavdzic_perica@yahoo.com (P.M.); branko0089@gmail.com (B.G.); iv.tomic@yahoo.com (I.T.); milos.sladojevic@gmail.com (M.S.); dr.koncar@gmail.com (I.K.); 2 Clinic for Vascular and Endovascular Surgery, Clinical Center of Serbia, 11000 Belgrade, Serbia; 3 Center for Radiology, University Clinical Center of Vojvodina, 21000 Novi Sad, Serbia; kokovic.tijana@gmail.com; 4 Medical Faculty, Military Medical Academy, Belgrade Defence University, 11040 Belgrade, Serbia; tomicdoc@gmail.com

**Keywords:** carotid artery stenosis, carotid plaque, multidetector computed tomography angiography (MDCTA), duplex ultrasonography (DUS), interobserver agreement, plaque morphology, plaque composition, stroke risk stratification, vascular imaging

## Abstract

*Background and Objectives*: Carotid artery stenosis has traditionally guided therapeutic decision-making; however, plaque morphology and composition are increasingly recognized as more reliable indicators of cerebrovascular risk than luminal narrowing alone. As imaging strategies shift toward vulnerability-based assessment, reproducibility of plaque characterization becomes essential for consistent clinical decision-making. This study aimed to evaluate interobserver agreement in carotid plaque assessment using multidetector computed tomography angiography (MDCTA) and to assess intermodality agreement with duplex ultrasonography (DUS). *Materials and Methods*: In this single-center study (January–September 2022), 50 patients with ≥60% internal carotid artery stenosis diagnosed by DUS (NASCET criteria), the majority of whom were asymptomatic (90%), were included. MDCTA examinations were independently analyzed by two radiologists, while DUS examinations were evaluated by a third observer. Plaque composition (lipid, fibrous, calcified), surface characteristics (regular, irregular, ulcerated), degree of stenosis, and plaque length were assessed. CT plaque characterization was based on Hounsfield unit (HU) thresholds (<50 HU lipid; 50–120 HU fibrous; >120 HU calcified). Interobserver agreement and intermodality agreement were calculated using Cohen’s kappa coefficient. *Results*: Good interobserver agreement was observed between the two MDCTA readers (κ = 0.751). Intermodality agreement between MDCTA and DUS was moderate (κ = 0.624 and κ = 0.595). Although significant differences were identified in 3 of 16 HU measurement points, no significant differences were found in overall plaque composition classification between MDCTA observers. DUS yielded significantly higher stenosis values (*p* = 0.007 and *p* = 0.005) and greater plaque length measurements (*p* < 0.0005) compared with MDCTA. Significant differences were also observed in plaque surface assessment between modalities (*p* = 0.044 and *p* = 0.033). *Conclusions*: MDCTA demonstrates good interobserver reproducibility for carotid plaque characterization, while intermodality agreement between MDCTA and DUS is moderate. Minor attenuation measurement differences do not significantly affect plaque classification; however, systematic intermodality differences in stenosis grading, plaque surface evaluation, and plaque length measurement should be considered in clinical decision-making.

## 1. Introduction

Carotid artery stenosis has traditionally been the principal indicator for therapeutic decision-making and follow-up in patients at risk for cerebrovascular events [1,2]. However, stenosis severity alone does not fully capture the pathophysiological complexity of atherosclerotic disease, and it has become increasingly clear that plaques with similar degrees of luminal narrowing can exhibit markedly different risks of rupture and subsequent ischemic events [3,4].

Current clinical decision-making in carotid artery disease is guided by international recommendations, including the 2023 guidelines of the European Society for Vascular Surgery (ESVS), which emphasize not only the degree of stenosis but also plaque morphology and imaging characteristics in determining indications for carotid endarterectomy or carotid artery stenting [5]. Imaging modalities therefore play a central role in identifying high-risk plaque features and guiding appropriate patient selection for intervention.

Plaque vulnerability is determined by morphological and compositional features such as a large lipid-rich necrotic core, thin fibrous cap, intraplaque hemorrhage, surface ulceration, and overall plaque burden, which may be more reliable predictors of index and recurrent cerebrovascular events than stenosis degree alone [3,6,7]. With advances in vascular imaging, assessment has shifted from purely quantifying luminal stenosis to detailed characterization of plaque features that confer instability and elevated stroke risk [3,4]. Besides others, duplex ultrasonography (DUS) and computed tomography angiography using multidetector scanners (MDCTA) can provide detailed morphological information regarding plaque surface characteristics, extent, and composition that are relevant to stroke risk stratification [5,8,9,10].

A significant challenge in clinical practice is the reproducibility of imaging findings, as subjective interpretation may vary between observers. Interobserver variability can significantly affect measurement consistency in both DUS and MDCTA studies, which in turn may influence clinical decision-making. Assessment of reproducibility is commonly evaluated by calculating interobserver agreement [11].

The aim of this study was to evaluate interobserver agreement in the measurement of carotid plaque characteristics using MDCTA measurements and DUS measurements, in order to assess the reproducibility of these image analysis between modalities and among individual observers.

## 2. Materials and Methods

This single-center study included 50 patients with carotid artery stenosis who were admitted to our institution between 3 January 2022, and 1 September 2022. During this period, patients underwent routine clinical evaluation, including duplex ultrasonography (DUS) and multidetector computed tomography angiography (MDCTA), based solely on clinical indications and not for research purposes. The study protocol was designed retrospectively after completion of patient inclusion. Ethical approval was obtained from the Ethics Committee of the Faculty of Medicine, University of Belgrade (Approval No. 1322/IX-5; approved on 22 September 2022). Following approval, all eligible patients were contacted and provided written informed consent for participation in the study and use of their clinical and imaging data. All study-related analyses were conducted only after ethics approval, in accordance with institutional and international ethical standards.

Patients were initially evaluated using DUS. Inclusion criteria were internal carotid artery stenosis ≥ 60%, as determined by DUS according to the North American Symptomatic Carotid Endarterectomy Trial (NASCET) criteria. Exclusion criteria included carotid artery stenosis < 60%, complete occlusion of the internal carotid artery, and non-atherosclerotic carotid disease. A total of 50 patients met the inclusion criteria and underwent further imaging with MDCTA.

DUS examinations were performed by an experienced radiologist using a Siemens Acuson P500 system with a 6–18 MHz linear probe, following standardized protocols for carotid artery assessment. The following plaque characteristics were evaluated: echogenicity according to the Gray–Weale classification (Class I–V), plaque morphology (lipid, fibrous, or calcified) [12], degree of stenosis (based on NASCET criteria), plaque surface (regular, irregular, or ulcerated), and plaque length measured in centimeters.

All MDCTA scans were performed using a Toshiba Aquilion ONE 640 scanner (Toshiba America Medical Systems, Inc., Tustin, CA, USA). A non-ionic iodinated contrast agent (Ultravist 370, Bayer HealthCare, Leverkusen, Germany) was administered intravenously at a volume of 110–130 mL and a flow rate of 4–6 mL/s. Imaging parameters included a 512 × 512 matrix, field of view 12–19 cm, tube current 180–210 mA, tube voltage 120 kV, and slice thickness of 1.6 mm. Axial images were reconstructed at 0.75 mm, and multiplanar reconstruction (MPR) and maximum intensity projection (MIP) images were generated for analysis.

Two experienced radiologists independently evaluated the MDCTA examinations (observer 1 and observer 2), while DUS examinations were assessed by a third experienced radiologist (observer 3). These designations were used consistently throughout the analysis.

Plaque composition on MDCTA was assessed using Hounsfield unit (HU) attenuation values according to established criteria (<50 HU for lipid, 50–120 HU for fibrous tissue, and >120 HU for calcified components). For each plaque, 16 regions of interest (ROI) were placed, and the dominant plaque component was defined based on the most frequently observed tissue type. Degree of stenosis was measured using NASCET criteria on axial images perpendicular to the vessel lumen. Plaque surface morphology was classified as regular, irregular, or ulcerated, and plaque length was measured in millimeters.

**Figure 1:**
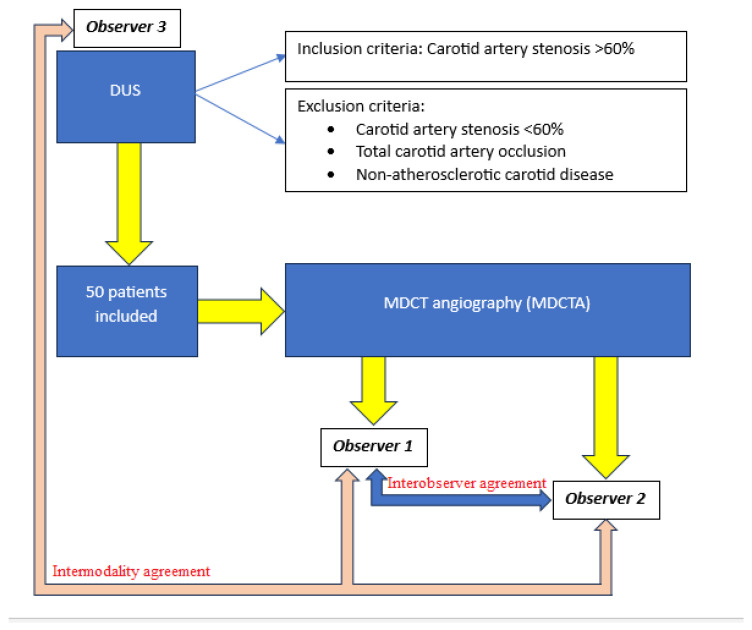
Study protocol–patient selection and flowchart.

The study protocol, including patient selection and the study flowchart, is summarized in Figure 1.

### Statistical Analysis

Statistical analysis was performed using IBM SPSS Statistics version 25. Continuous variables are presented as mean ± standard deviation. Paired sample *t*-tests and one-way repeated-measures ANOVA were used to assess differences between measurements. Statistical significance was set at *p* < 0.05. Interobserver agreement (between observer 1 and observer 2 for MDCTA) and intermodality agreement (between MDCTA and DUS assessments) were evaluated using Cohen’s kappa coefficient.

## 3. Results

### 3.1. Patient Characteristics

A total of 50 patients (32 males and 18 females) with a mean age of 68.9 years (range: 40–82 years) were included in the study. The majority of patients were asymptomatic (90%), while 10% presented with symptoms related to carotid artery disease.

### 3.2. Interobserver and Intermodality Agreement

Interobserver agreement for MDCTA-based plaque assessment between observer 1 and observer 2 was good (κ = 0.751). Intermodality agreement between MDCTA and DUS was moderate, with κ = 0.624 for observer 1 (MDCTA) versus observer 3 (DUS), and κ = 0.595 for observer 2 (MDCTA) versus observer 3 (DUS).

### 3.3. HU Measurements on MDCTA

Comparison of Hounsfield unit (HU) measurements between the two MDCTA observers demonstrated statistically significant differences at three of sixteen measurement points: measurement 2 (*p* = 0.043), measurement 8 (*p* = 0.020), and measurement 13 (*p* = 0.025), where observer 2 reported higher attenuation values. No statistically significant differences were observed in the remaining thirteen measurement points (Table 1).

### 3.4. Plaque Composition on MDCTA

No statistically significant differences were observed between observer 1 and observer 2 in the assessment of plaque composition, including lipid (*p* = 0.118), fibrous (*p* = 0.193), and calcified (*p* = 0.684) components (Table 2).

### 3.5. Ultrasound Plaque Classification

According to the Gray–Weale classification of plaque echogenicity on DUS, most patients were categorized as Class III (34%) and Class IV (34%), corresponding to predominantly fibrocalcified and calcified plaques. No plaques were classified as Class I or Class V (Table 3).

Morphological classification based on DUS demonstrated a predominance of fibrous (46%) and calcified (48%) plaques, while lipid-rich plaques were less common (6%) (Table 4).

### 3.6. Comparison of Stenosis Degree, Plaque Surface, and Plaque Length

Statistically significant differences were observed between MDCTA and DUS measurements of stenosis degree, plaque surface, and plaque length (Table 5).

DUS yielded significantly higher stenosis values compared with MDCTA measurements by observer 1 (*p* = 0.007) and observer 2 (*p* = 0.005).

Intermodality comparison of plaque surface assessment revealed significant differences between DUS and MDCTA for both observer 1 (*p* = 0.044) and observer 2 (*p* = 0.033), with DUS more frequently identifying regular plaque surfaces and fewer ulcerations.

Plaque length measurements were significantly greater when assessed by DUS compared to MDCTA (*p* < 0.0005 for both comparisons).

No statistically significant differences were observed between observer 1 and observer 2 (MDCTA) in the assessment of stenosis degree (*p* = 0.253), plaque surface (*p* = 0.081), or plaque length (*p* = 0.791).

Distribution of plaque surface characteristics across modalities is presented in Table 6.

### 3.7. Comparison of Plaque Morphology Among Three Observers

Repeated-measures ANOVA demonstrated no statistically significant differences in overall plaque morphology classification among observer 1 (MDCTA), observer 2 (MDCTA), and observer 3 (DUS) (F = 0.048, *p* = 0.954) (Table 7).

## 4. Discussion

The findings of this study provide insight into the interobserver agreement and methodological challenges involved in assessing carotid plaque characteristics using DUS and MDCTA. These results underscore both the utility and the variability of these imaging techniques in evaluating plaque morphology, stenosis, and vulnerability, critical factors in stratifying stroke risk in patients with carotid atherosclerosis.

The present study evaluated interobserver agreement in the assessment of carotid plaque characteristics using MDCTA and compared these findings with DUS measurements. The results demonstrate good agreement between two independent MDCTA observers and moderate agreement between MDCTA and DUS assessments. These findings support the reproducibility of CT-based plaque evaluation while also highlighting intrinsic differences between imaging modalities that may influence clinical interpretation.

A kappa value of 0.751 between the two MDCTA observers indicates good agreement according to widely accepted interpretation criteria [13]. This finding confirms that, when standardized acquisition protocols and window settings are applied, MDCTA provides reliable and reproducible measurements of plaque morphology, degree of stenosis, plaque surface characteristics, and length.

The clinical importance of reproducibility in carotid imaging can not be overstated. Treatment decisions, including carotid endarterectomy or carotid artery stenting, often rely on imaging findings. Landmark trials such as NASCET and European Carotid Surgery Trial (ECST) established the benefit of intervention based primarily on stenosis severity. However, contemporary evidence suggests that plaque composition and morphology may be more closely associated with stroke risk than luminal narrowing alone [1,2,3]. Consequently, variability in imaging interpretation may directly influence patient management strategies.

Our results are complementary to previous studies demonstrating high diagnostic accuracy and reproducibility of MDCTA for carotid stenosis grading compared with digital subtraction angiography [14,15]. MDCTA offers excellent spatial resolution and the ability to evaluate both luminal narrowing and plaque surface irregularities, including ulcerations, which are recognized markers of plaque vulnerability [16]. The good interobserver agreement observed in our study further supports MDCTA as a dependable modality in routine clinical practice. Although statistically significant differences were observed in 3 of 16 HU measurement points, these variations did not result in significant differences in overall plaque composition classification between observers. This finding suggests that small regional attenuation discrepancies may occur due to subtle differences in ROI placement, partial volume effects, or beam-hardening artifacts, but they do not substantially alter dominant plaque categorization.

CT attenuation–based classification of plaque components (<50 HU lipid, 50–120 HU fibrous, >120 HU calcified), originally described by Schroeder et al., remains widely used for plaque characterization [13]. Several subsequent investigations have confirmed that lower attenuation values correlate with lipid-rich necrotic cores, whereas higher values correspond to fibrous or calcified tissue [17,18].

However, attenuation thresholds are not absolute and may be influenced by scanner type, reconstruction algorithms, and contrast timing [19]. This may explain the minor measurement discrepancies observed in our study. Importantly, despite these variations, no significant interobserver difference was found in overall plaque morphology classification on MDCTA, further supporting the robustness of CT-based plaque assessment.

It is important to distinguish interobserver agreement, which reflects consistency between readers within the same imaging modality, from intermodality agreement, which reflects differences between distinct imaging techniques. These represent fundamentally different sources of variability and should be interpreted accordingly when applying imaging findings to clinical decision-making. Agreement between MDCTA and DUS was moderate (κ = 0.624 and κ = 0.595). This level of agreement is expected given the inherent differences between modalities. DUS evaluates both morphological and hemodynamic parameters, whereas MDCTA primarily provides anatomical information. A significant finding of our study was that DUS contributed higher measurements of stenosis severity compared to MDCTA. This may be explained by the fact that Doppler ultrasound calculates stenosis based on velocity criteria and flow dynamics, which can sometimes overestimate stenosis in the presence of turbulent flow or contralateral disease [20]. In contrast, MDCTA provides direct visualization of luminal narrowing based on anatomical measurement. The discrepancy observed in our cohort is consistent with prior studies reporting systematic differences between ultrasound and CT measurements [21].

Similarly, plaque length was significantly greater when measured by DUS compared to MDCTA. Ultrasound may overestimate plaque extent due to longitudinal insonation and difficulty in defining plaque margins precisely, especially in heavily calcified lesions. Conversely, CT allows multiplanar reconstruction, which may provide a more anatomically precise but sometimes shorter measurement depending on slice orientation [22].

Regarding plaque surface characterization, DUS most frequently identified regular surfaces, while ulcerated plaques were less commonly reported compared with CT. MDCTA, due to its high spatial resolution and multiplanar capabilities, may be superior in detecting ulcerations, which have been associated with increased embolic risk [4,16]. This difference likely contributed to the statistically significant intermodality variability observed.

Ultrasound analysis demonstrated predominance of fibrous and calcified plaques (94% combined), with absence of purely lipid-rich (Class I) plaques. This distribution may reflect the clinical characteristics of the study population, in which 90% of patients were asymptomatic. Calcified and fibrocalcified plaques are generally considered more stable compared to lipid-rich plaques [5,6].

Several studies have demonstrated that echolucent plaques (Gray–Weale Class I and II) are associated with higher stroke risk, whereas echogenic and calcified plaques are more stable [23]. The absence of highly echolucent plaques in our cohort may partially explain the predominance of moderate-to-high stenosis without high symptomatic burden. Although subgroup analysis of symptomatic and asymptomatic patients would be of clinical interest, it was not performed due to the small proportion of symptomatic patients (10%), which limits statistical power.

This study has several important clinical implications. First, MDCTA shows strong interobserver reproducibility, affirming its reliability for assessing plaque morphology. The moderate agreement between MDCTA and DUS indicates that these techniques are complementary rather than interchangeable. Clinical decision-making should account for systematic differences in stenosis grading and plaque length measurements between the two modalities. Additionally, minor variability in Hounsfield Unit (HU) measurements does not significantly impact plaque classification, highlighting the robustness of attenuation-based categorization when standardized protocols are used. Given the growing emphasis on plaque vulnerability rather than stenosis alone, reproducible imaging biomarkers are essential. Combined evaluation using DUS (hemodynamics) and MDCTA (morphology and surface characterization) may provide the most comprehensive assessment of stroke risk.

### Study Limitations

Several limitations should be acknowledged. First, the study included a relatively small sample size (*n* = 50), which may limit generalizability. Second, only a single observer evaluated DUS examinations, precluding assessment of interobserver variability within ultrasound and representing an additional limitation. Third, histopathological correlation was not available; therefore, imaging-based plaque characterization could not be validated against surgical specimens. Additionally, HU thresholds used for plaque classification, although widely accepted, may vary across scanners and reconstruction algorithms. Finally, subgroup analysis between symptomatic and asymptomatic patients was not performed due to the small number of symptomatic cases.

Future studies with larger cohorts, multiple ultrasound observers, and histopathological correlation would further clarify intermodality reproducibility, diagnostic accuracy, and the generalizability of imaging-based plaque characterization across both symptomatic and asymptomatic patients.

## 5. Conclusions

In conclusion, MDCTA shows good interobserver agreement in assessing carotid plaque characteristics, while agreement between MDCTA and DUS is moderate; minor differences in attenuation measurements do not significantly influence plaque morphology classification, although systematic differences in stenosis grading, plaque surface evaluation, and plaque length measurement between the two modalities should be acknowledged. These findings support the complementary use of DUS and MDCTA in carotid artery disease assessment, emphasize the importance of standardized imaging protocols to ensure reproducibility and reduce bias, and suggest that dual-modality approaches and the development of integrated or standardized techniques could improve stroke risk stratification and enable more tailored management of patients with carotid atherosclerosis.

## Figures and Tables

**Table 1:** Differences in the measurement of HU units of plaques on MDCTA between observers.

	Observer 1 (*n* = 50)	Observer 2 (*n* = 50)	T	*p*
	Mean			
Measurement 1	446.96 ± 530.38	491.26 ± 613.94	−0.661	0.511
Measurement 2	396.58 ± 454.59	522.48 ± 599.22	−2.075	0.043 *
Measurement 3	463.32 ± 484.23	492.94 ± 622.46	−0.480	0.633
Measurement 4	438.30 ± 489.75	490.66 ± 573.09	−0.799	0.428
Measurement 5	415.66 ± 526.52	407.70 ± 556.64	0.148	0.883
Measurement 6	340.68 ± 428.88	420.42 ± 513.59	−1.120	0.268
Measurement 7	381.92 ± 527.06	381.33 ± 581.48	0.010	0.992
Measurement 8	341.30 ± 508.74	511.62 ± 651.97	−2.404	0.020 *
Measurement 9	336.34 ± 434.97	437.34 ± 558.81	−1.665	0.102
Measurement 10	427.10 ± 553.76	513.84 ± 629.41	−1.199	0.236
Measurement 11	482.42 ± 505.57	579.14 ± 599.43	−1.411	0.165
Measurement 12	451.98 ± 475.57	424.10 ± 507.08	0.400	0.691
Measurement 13	349.38 ± 417.68	489.78 ± 607.92	−2.318	0.025 *
Measurement 14	392.52 ± 468.49	541.50 ± 574.05	−1.866	0.068
Measurement 15	411.02 ± 517.42	433.06 ± 507.68	−0.470	0.640
Measurement 16	417.84 ± 498.50	457.14 ± 595.95	−0.653	0.517

* Statistical significance at the level of 0.05.

**Table 2:** Differences in the representation of plaque morphology on MDCTA between observers.

	Observer 1 (*n* = 50)	Observer 2 (*n* = 50)	T	*p*
	Mean			
Lipid	2.52 ± 2.93	3.02 ± 3.44	−1.589	0.118
Fibrous	4.98 ± 3.63	4.56 ± 3.47	1.320	0.193
Calcified	8.50 ± 4.59	8.42 ± 4.47	0.409	0.684

Statistical significance at the level of 0.05.

**Table 3:** Gray-Weale classification of plaque echogenicity based on DUS.

	f	%
Class I	0	0
Class II	16	32
Class III	17	34
Class IV	17	34
Class V	0	0
Total	50	100

**Table 4:** Morphological classification of plaque based on DUS.

	f	%
Lipid	3	6
Fibrous	23	46
Calcified	24	48
	50	100

**Table 5:** Interobserver differences in measuring stenosis degree, plaque area, and plaque length on MDCTA and DUS.

	Observer 1 (*n* = 50)	Observer 2 (*n* = 50)	Observer 3 (*n* = 50)	F	*p*
	Mean				
Degree of stenosis (%)	79.30 ± 9.63	77.60 ± 10.74	83.00 ± 9.14	5.817	0.005 *
Plaque surface	2.14 ± 0.75	2.18 ± 0.71	1.92 ± 0.77	4.039	0.024 *
Plaque length (mm)	1.74 ± 0.57	1.79 ± 0.61	1.90 ± 0.49	13.286	<0.0005 *

* Statistical significance at the level of 0.05.

**Table 6:** Plaque area on MDCTA and DUS examination.

	Observer 1		Observer 2		Observer 3	
	f	%	f	%	F	%
Regular	11	22	9	18	17	34
Irregular	21	42	23	46	20	40
Ulcerated	18	36	18	36	13	26

**Table 7:** Interobserver differences in plaque morphology assessment on MDCTA and DUS.

	Observer 1 (*n* = 50)	Observer 2 (*n* = 50)	Observer 3 (*n* = 50)	F	*p*
	Mean				
Plaque morphology	2.44 ± 0.73	2.44 ± 0.76	2.42 ± 0.60	0.048	0.954 *

* Statistical significance at the level of 0.05.

## Data Availability

The original contributions presented in this study are included in the article. Further inquiries can be directed to the corresponding author(s).
